# Social Capital as a Moderator of the Relationship Between Perceived Discrimination and Alcohol and Cannabis Use Among Immigrant and Non-immigrant Adolescents in Israel

**DOI:** 10.3389/fpsyg.2018.01556

**Published:** 2018-09-05

**Authors:** Sophie D. Walsh, Tanya Kolobov, Yossi Harel-Fisch

**Affiliations:** ^1^Department of Criminology, Bar-Ilan University, Ramat Gan, Israel; ^2^International Research Program on Adolescent Well-Being and Health, School of Education, Bar-Ilan University, Ramat Gan, Israel

**Keywords:** discrimination, alcohol and cannabis use, social capital, adolescent immigrants, Israel, parental monitoring, teacher support, peer support

## Abstract

Literature highlights the relationship between perceived discrimination and frequency and severity of alcohol and cannabis use. One mechanism for explaining this is the nature of perceived discrimination as a potentially traumatic interpersonal stressor, which can lead to the depletion of social and personal resources. Within a Recovery Capital (RC) framework, the current study explores whether the existence of social capital in the form of parental monitoring, friend and teacher support can buffer the relationship between perceived discrimination and alcohol and cannabis use among immigrant and non-immigrant adolescents, by replenishing the depleted resources. The study included a representative sample of 8,598 students in Israel, aged 11–18, from the Health Behaviors of School Aged Children (HBSC) 2013–2014 data: 1503 immigrant adolescents from the Former Soviet Union [FSU] (*N* = 955) and Ethiopia (*N* = 548) and 7086 non-immigrants. Results confirmed that perceived discrimination was positively related to substance use; all three forms of social capital were negatively related to alcohol and cannabis use and moderated the relationship between perceived discrimination and substance use, with the exception of friend support in the case of alcohol use. When all three social capital measures were included together, the adult social capital measures were significant predictors of substance use. Results suggest that levels of social capital, especially as provided by parents and teachers, can help young people, both immigrant and majority group adolescents, to cope with perceived discrimination.

## Introduction

Alcohol use in adolescence is a matter of public health concern due to its interrelation to additional risk behaviors, the tendency for alcohol use to persist into adulthood and the impact it can have on the adolescent brain and health in general (Marshall, 2014). Alcohol use is the leading factor for cause-specific disability-adjusted life years (DALYs) for young people aged 10–24 years (Gore et al., 2011). Public health concerns over adolescent cannabis or marijuana use (Hopfer, 2014) have focused on its relationship with many diverse outcomes such as impaired cognitive function (Tapert et al., 2008), an impact on current functioning, such as school attainment (Lynskey and Hall, 2000) and its impact on later life outcomes (Fergusson and Boden, 2008) such as unemployment, welfare dependence and relationship satisfaction.

Among the many predictors of alcohol and cannabis use among adolescents, increasing literature has pointed to the relationship between perceived discrimination and substance use among majority and minority adolescents (Gibbons et al., 2004; Otiniano Verissimo et al., 2014; Acosta et al., 2015; Unger et al., 2016). A recent study in Israel (Walsh et al., 2018) pointed to the significant relationship between perceived discrimination and substance use (alcohol and cigarettes) among first and second generation immigrant adolescents in Israel. Despite the relationship between perceived discrimination and substance use, literature to date has not examined potential resources which could buffer or moderate the impact of perceived discrimination on young people. The current study uses a framework of recovery capital to examine social relationships (social capital) as moderating the relationship between perceived discrimination and alcohol and cannabis use among a large representative sample of immigrant and non-immigrant adolescents in Israel.

### Perceived Discrimination as a Traumatic Stressor

According to a bio-psycho-social model, perceived discrimination is an interpersonal stressor (Clark et al., 1999). Discriminatory behaviors may be internalized and give a message to a young person that the society does not accept them, and that their opportunities for success and achieving are limited (Motti-Stefanidi et al., 2012). They can awaken feelings of rejection, helplessness and despair which can impact negatively on their well-being (Jasinskaja- Lahti et al., 2006). Basing his work on Carlson’s (1997) conceptualization of traumatic stress, Carter (Carter et al., 2005; Carter, 2007; Carter and Forsyth, 2010) suggests that racism or perceived discrimination can be experienced not just as a stressor, but at times can take on dimensions of trauma. Carter (2007) suggests that race-based traumatic stress can lead to symptoms of intrusion (re-experiencing), avoidance (numbing) and increased arousal or vigilance, characteristic of PTSD, and that definitions of trauma need to extend beyond real or imagined physical threat to emotional and psychological threat. According to Carlson (1997), traumatic stress involves three components: the subjective appraisal or perception of an event as negative, the event experienced as sudden and without warning and it’s experience as uncontrollable. All of these criteria can be part of the experience of perceived discrimination. The experience of trauma can also lead to a depletion of social and personal resources (Hobfoll, 1991), meaning that the existence of social capital can be particularly significant in recovery. The response to trauma also depends on additional factors such as the individual’s age, physical condition, severity (e.g., number of times and intensity), previous life-time events and whether the social context is supportive or not. In an extensive review of studies examining the relationship between racism and traumatic stress (Carter, 2007), racial stress has been found to be related to negative physiological (Harrell et al., 2003), and psychological (Pieterse et al., 2010) health outcomes, maladaptive coping behaviors and poor relationship quality (Murry et al., 2001).

Among adolescents, perceived discrimination has been consistently shown to predict lower levels of adaptation and well-being among immigrant and minority adolescents (Noh and Kaspar, 2003; Sabatier and Berry, 2008; Berry and Sabatier, 2010; Motti-Stefanidi et al., 2012). Adolescence is a developmental period, in which positive peer relations are pivotal in predicting adolescent health (Viner et al., 2012) and in which the feelings of social and interpersonal rejection and ostracism that can be experienced as a result of perceived discrimination (Smart Richman and Leary, 2009) may lead to intense psychological distress. Perceived discrimination has been found to be related to lower psychological well-being (Jasinskaja- Lahti et al., 2006; Davis et al., 2016) and school performance (Helms, 2003; English et al., 2016) and higher levels of violent behavior (Williams et al., 2014). Perceived discrimination has been related to substance use, including smoking, alcohol and marijuana use (Okamoto et al., 2009; Unger et al., 2014) among Latino youth, and has also been shown to be a significant predictor of the severity of alcohol use (Cano et al., 2015b) and increased drunkenness over time (Schwartz et al., 2015). In addition, a recent study on trajectories of perceived discrimination and their relationship to substance use, found that the group with high and stable experiences of perceived discrimination showed the highest levels of both last month alcohol and cigarette use (Unger et al., 2016). However, despite recent research, the impact of perceived discrimination on adolescent well-being is still understudied (Davis et al., 2016), especially among populations outside of the United States.

### Recovery Capital, Perceived Discrimination and Substance Use

Recovery Capital (RC) (Granfield and Cloud, 1999) as a comprehensive paradigm for understanding recovery from substance abuse, outlines key personal and social resources which the individual can draw upon to enable cessation from substance use and encourage reintegration into the community (Chen, 2017; Connolly and Granfield, 2017). Formulation of RC rests on the understanding that the ability of an individual to desist from substance use is related to a range of factors including the environmental context they are in, the personal characteristics they possess and the range of resources available to them. In particular, four forms of capital (Cloud and Granfield, 2008) have been identified: social, physical (e.g., economic or financial), human (attributes such as knowledge, skills, education and mental health) and community/cultural (e.g., community support institutions, cultural norms, values, beliefs and perceptions which a person to harness) capital. In the current study we focus on social capital. Social or family capital encompasses family and social relationships that are supportive of the individual desisting from substance use (White and Cloud, 2008). Social capital (Connolly and Granfield, 2017) includes social networks, peer and family support and can provide friendship, social ties, empathy and caring. While, to date, little research has focused on RC among adolescents, Cloud and Granfield (2008) suggest that age can impact on the amount of recovery capital (both positive and negative) that one can accumulate. On the one hand, adolescents have not had substantial time to accumulate large amounts of RC; yet on the other hand, they may not have had the same negative life experiences that can have depleted their RC.

Previous literature has highlighted the role of social support, especially parental, in decreasing adolescent substance use (Wills et al., 1996, 2004). In this particular study, three types of social capital are examined: parental monitoring, teacher support, and friend support. They were chosen for their range (parent, peer, and teacher) of forms of social support which have been empirically found to relate to substance use. The relationship between adolescent parent and peer relationships and involvement in substance use has been well-researched. Elevated levels of positive parental relationships, in particular parental monitoring (Abar et al., 2015; Mynttinen et al., 2017) have been found to be negatively related to lower substance use (Lac and Crano, 2009; Thompson et al., 2015; Donaldson et al., 2016). In their analysis of parental monitoring, Stattin and Kerr (2000) suggest that the, far from being about parental control or even parental activity, parental monitoring is about child disclosure and about the existence of a parental–child relationship in which positive communication and support channels mean that the young person shares with their parents what they are doing. However, research suggests that parent and peer relations may differently impact on adolescent substance use. Research on peer relationships and substance use has tended to focus on the positive relationship between substance using or deviant peers and adolescent alcohol and cannabis use (Handren et al., 2016; Korhonen et al., 2008; Wang et al., 2017; Cambron et al., 2018). However, few studies have examined the role of peer support (Wills et al., 2004), i.e., the extent to which the young person has close and supportive peer relationships in general. In the current study, we would suggest that in the relationship between perceived discrimination and substance use, where the young person may feel a sense of alienation as a result of perceived discrimination (Walsh et al., 2017), peer support may be a critical buffer.

Few studies have examined the relationship between adolescent substance use and school (Demaray and Malecki, 2002; Chong et al., 2006). Teacher support has been found to be related to lower levels of drug use (Larusso et al., 2008). School perceptions (including feelings of social connectedness, teacher caring and respect) have been found to be related to smoking, drinking, violence and truancy (see Larusso et al., 2008 for a review). Positive relationships with non-family adult mentors (mainly teachers or guidance counselors) are related to lower levels of smoking, depressive symptoms and suicide ideation, risk taking, violence and gang membership (DuBois and Silverthorn, 2005a,b).

In the current study we examine these three relationships as potential buffers (moderators) of the relationship between perceived discrimination and alcohol use. As such, the study connects between the literature on RC and literature on traumatic stress and post-traumatic growth (Tedeschi and Calhoun, 2004). Posttraumatic growth (PTG) is the perceived positive change that the individual experiences after struggling with challenging life crises. According to Calhoun and Tedeschi (1999), Tedeschi and Calhoun (2004), this involves internal cognitive and emotional changes which happen following an event which challenges core beliefs about the world. Perceived discrimination can challenge the individual’s views of him/herself (e.g., perspectives of worth and value) and of society (e.g., as accepting and allowing opportunities) (Jasinskaja- Lahti et al., 2006; Walsh et al., 2017). A key element in enabling the process of PTG is the existence of social support (Tedeschi and Calhoun, 2004). Social support of others can allow the individual build new narratives or schema. Mutual support is enabled through talking with others who have experienced similar experiences. Through sharing experiences with others, an intimacy can be created, emotional experiences shared and others can offer perspectives which can be integrated into schema change. Within the context of perceived discrimination, parental, peer and teacher support can enable the young person to build new schema or narratives which can facilitate their coping with the negative emotions aroused by the discriminatory behaviors. Scarce literature suggests that social support (parent, peer and school) can indeed buffer the relationship between perceived discrimination on academic well-being (DeGarmo and Martinez, 2006), but little literature has examined the buffering role of social capital on the relationship between perceived discrimination and substance use.

Within a motivational model of alcohol use (Cooper et al., 1995), a self-medication explanation (Virtanen et al., 2015) or a stress-coping model (Wills and Cleary, 1995) substance use can be a means by which young people express and try to cope with their psychological distress. In line with a model of race related traumatic stress (Carter and Forsyth, 2010) and a framework of RC (Granfield and Cloud, 1999; Cloud and Granfield, 2008) and PTG we suggest that social relations can help young people to make sense and process the potentially traumatic experiences of perceived discrimination (Tedeschi and Calhoun, 2004), counteract experiences of interpersonal rejection (Smart Richman and Leary, 2009), restore depleted resources (Hobfoll, 1991) and strengthen their self-esteem (Umana-Taylor and Updegraff, 2007). As such they can weaken the effects of perceived discrimination on the young person and reduce the need for the young person to turn to substance use to moderate painful emotions.

### Immigrant Adolescents in Israel

The current study takes place in Israel and examines two groups of immigrant adolescents, Ethiopian-heritage and FSU-heritage, in Israel, alongside a larger group of non-immigrant adolescents. Israel is a country with complex ethnic diversity in which issues of prejudice and perceived discrimination, over and above social status, are relevant for the majority non-immigrant population due to historical ethnic divisions between European and North African Jewish immigrants (Schwartz et al., 1991) making the study of the relationships between perceived discrimination, RC and substance use relevant for both the immigrant and non-immigrant population. Statistics report 60,000 immigrant adolescents aged 12–17 from the Former Soviet Union (FSU) (35% first generation) and 17,900 Ethiopian-heritage adolescents (45% first generation) (Kahan-Strawczynski et al., 2012), the two largest immigrant groups to Israel in recent years, approximately 19% of all 12–17 year olds in Israel^1^. The wave of immigrants from the FSU following 1990 took place after the breakup of the FSU, in the socio-economic crisis and instability that ensued (Remennick, 1999). FSU-heritage adolescents came with high levels of education and human capital and studies have documented impressive levels of employment and integration (Amit, 2012; Remennick, 2012). Yet they have been subject to discrimination on the basis of their perceived symbolic and realistic threat (Tartakovsky and Walsh, 2016) and questioned Jewish status (Remennick, 2012). Studies show that adolescents from the FSU feel high levels of alienation in Israel, more so than Ethiopian adolescents (Kahan-Strawczynski et al., 2013), which may be related to the different motivations behind the immigration from the FSU which was largely for economic reasons (Al-Haj, 2002) and the immigration from Ethiopia which was largely for ideological reasons (Amit, 2012). Today, immigrants from the FSU make up 11% of the Jewish population (9% of the population as a whole) making them a significant minority.

The Ethiopian Jewish community came to Israel with a deep Jewish identity and a rich culture and heritage, with a prominence of strong social networks and an emphasis on the extended family and values of respect (Schwarz, 2016). However, on coming to Israel, difficulties in integration resulted from deep cultural differences (Kaniel, 1990; Tannenbaum, 2008), such as the transition from poor rural living to an urban society, significant illiteracy and a more patriarchal culture with religious and community leaders acting as high authority (Kurman and Ronen-Eilon, 2004), as well as racism and discrimination on the basis of skin color (Offer, 2007). Two major waves of immigration took place in 1984 and 1991, with continued immigration into the 21st century. Research shows the overall disadvantaged socio-economic status of the Ethiopian community, as well as substantial gaps in educational and occupational attainment (Offer, 2004, 2007). In 2015, waves of protest among the Israeli born (“second generation”) Ethiopian-heritage young people, following the video-taped attack on a young Ethiopian man by police, highlighted the still existing feelings of racism, disadvantage and unequal opportunities that Ethiopian-heritage young people in Israel experience today, despite their full participation in the army and the work force.

### The Current Study

**FIGURE 1:**
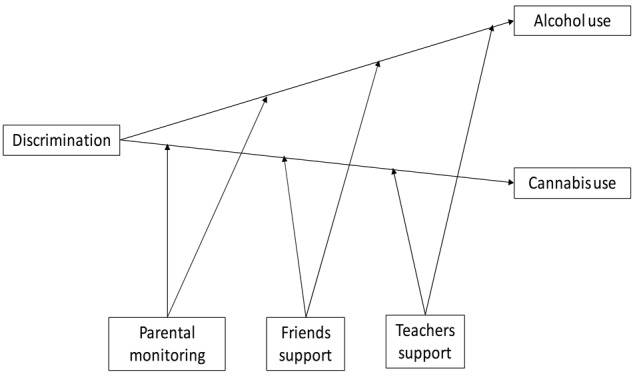
Conceptual model.

The current study focuses on social capital and asks whether three forms of social capital (parental monitoring, peer and teacher support) can buffer (moderate) the relationship between perceived discrimination and alcohol (drunkenness and binge drinking) and cannabis use among immigrant and non-immigrant adolescents (see **Figure 1** for conceptual model). The study hypothesized that (H1) perceived discrimination would be positively related to levels of alcohol and cannabis use; (H2) higher levels of parental monitoring, friend and teacher support would be related to lower levels of alcohol and cannabis use; (H3) higher levels of parental monitoring, friend support and teacher support would moderate (weaken) the relationship between perceived discrimination and alcohol and cannabis use (see **Figure 1** for conceptual model). In addition, due to the complex ethnically diverse nature of Israeli society (Schwartz et al., 1991) and perceived discrimination perceived by groups within the non-immigrant population as well as the immigrant population, we anticipated that the model would hold for both immigrant and non-immigrant adolescents, but that due to their difference in skin color (Offer, 2007) and recent immigration status, culture gaps and family acculturation difficulties (Walsh et al., 2010), immigrant adolescents from Ethiopia and the FSU would report higher levels of perceived discrimination (especially the Ethiopian adolescents) and lower levels of social capital.

## Materials and Methods

### Sample and Procedure

This study uses Israeli data from the 2013–2014 HBSC-WHO cross-national survey conducted among children aged 11–17. The HBSC is a school-based survey of adolescent health behaviors and psychosocial determinants carried out among representative samples of school aged children every 4 years, using an international standardized methodological protocol (Roberts et al., 2009; Currie et al., 2012), involving standardized procedures for sampling and translation of items (see Currie et al., 2014 for full protocol details). Although pupils at both Hebrew and Arabic speaking schools participated in the 2013–2014 Israeli study, questions around immigration were only asked in the Hebrew speaking schools. The study included a representative sample of 8,598 school students from 6th (33.7%), 8th (23.9%), and 10th–12th (42.5%) grades in Israel. We focused on three groups of adolescents: 1,503 immigrant adolescents (45.2% male) from the FSU and Ethiopia: 955 FSU-heritage adolescents (47.4% boys), 548 Ethiopian-heritage adolescents (42.4% boys) and 7086 non-immigrants (49.4% boys)^2^.

In order to ensure a representative sample, according to the international HBSC protocol (Currie et al., 2014) the Ministry of Education’s list of schools was used. Classrooms were randomly sampled (90% class-room response) and for each sampled school an additional class was also randomly sampled. All students in sampled classrooms present were included (>95% pupil response). The research protocol received approval from ethics committees of the Israeli Ministry of Education and Bar-Ilan University. In line with the Ethical committees, passive parental consent was obtained (letters were sent home to parents informing them of the study and requesting them to opt out if they preferred).

### Measures

#### Immigrant Status

Adolescents were asked three separate questions as to where they and each of their parents were born: “In which country were you/your mother/your father born?” [(1) Israel; (2) FSU; (3)Ethiopia; (4) An English speaking country; (5) a European country; (6) South America; (7) Other]. Former research indicates that children as young as 11 years old provide valid responses to these questions (Nordahl et al., 2011). In line with former research (Stevens et al., 2015) adolescents were considered immigrants if they or if at least one of their parents was born abroad.

#### Perceived Discrimination

Perceived discrimination was measured by three items developed by Phinney et al. (1998): “How often do the following people relate to you in a way which is negative or unfair because of your background”: teachers, pupils at school, children outside of school [(1) never, (2) rarely, (3)sometimes, (4) often, (5) almost always]. An overall perceived discrimination index combining all three items was created (Cronbach alpha FSU/Ethiopian/non-immigrant = 0.75/0.78/0.82).

#### Social Capital

Social capital was measured by three variables: parental monitoring, teachers’ support and friends’ support.

#### Parental Monitoring

Parental monitoring as assessed by the Parental Monitoring scale (Brown et al., 1993) which is created as the mean of 5 items: (1) your parents know who your friends are; (2) your parents know how you spend your money; (3) your parents know where you go after school; (4) your parents know where you go out at night; (5) your parents know what you are doing in your spare time. The values ranged from (1) Don’t know at all to (4) Know everything. The reliability for the monitoring scale was 0.69 for Ethiopians, 0.71 for FSU immigrants and 0.68 for non-immigrants.

#### Teacher Support

Teacher support was assessed using the amended Teacher Support Scale (Torsheim et al., 2000). The scale includes three question items: “I feel that my teachers accept me as I am,” “I feel that my teachers care about me as a person” and “I feel a lot of trust in my teachers.” Response options ranged from 1 – “very strongly disagree” to 5 – “strongly agree” for both teacher and classmate scales. Empirical research from a number of European countries (Torsheim et al., 2000) confirmed test–retest reliability and measurement invariance across countries. (Cronbach alpha FSU/Ethiopian/non-immigrant = 0.88/0.88/0.90).

#### Friends Support

Friends support was measured using the Multidimensional Scale of Perceived Social Support (MSPSS) (Zimet et al., 1988). The scale uses four questions: “My friends really try to help me,” “I can count on my friends when things go wrong,” “I have friends with whom I can share my joys and sorrows,” and “I can talk about my problems with my friends.” Response options range from 1 – “very strongly disagree” to 7 – “very strongly agree.” The MSPSS has been well-validated and used in multiple studies (Zimet et al., 1988, 1990) and across different cultural contexts (Edwards, 2004; Ng et al., 2010). (Cronbach alpha FSU/Ethiopian/non-immigrant = 0.91/0.91/0.91).

#### Alcohol Use

To build the resulting variable of “Alcohol use,” we used 5 alcohol-related items: (1) Drunk ever: “Have you ever had so much alcohol that you were really drunk?” (1-Never; 2-once; 3- 2–3 times; 4- 4–10 times; 5- more than 10 times); (2) Drunk in last 30 days: “Have you had so much alcohol that you were really drunk in the last 30 days?” (1-Never; 2-once; 3- 2–3 times; 4- 4–10 times; 5- more than 10 times); (3) Drinking before age 14: “At what age did you drink alcohol for the first time (more than a few sips)?”; (4) Drunk before 14: “At what age did you get drunk for the first time?” (5) Binge drinking in last 30 days: “In the past 30 days how many times have you drunk five drinks of alcohol or more within a period of a few hours?” (1- never; 2- not in the past month; 3- once; 4- twice; 5- three times; 6- four times or more). HBSC items on drunkenness and binge drinking have been well used and found to have good predictive and criterion validity (Kuntsche et al., 2011). For each question a dichotomous variable was created in order to identify adolescents involved in problematic alcohol use and due to the skewed nature of the distribution. A variable of alcohol use was calculated as sum of all alcohol-related items. Cronbach alpha (Ethiopian/FSU/non-immigrants = 0.79/0.72/0.73). The resulting values ranged from 0 to 5.

#### Cannabis Use

Cannabis use was measured as the number of times that the respondent reported using cannabis/marijuana ever. Responses were (1) ‘Never,’ (2) ‘1–2 times,’ (3) ‘3–5 times,’ (4) ‘6–9 times,’ (5) ‘10–19 times,’ (6) ‘20–39 times,’ and (7) ‘40 times or more.’ It is important to note, that due to ethical restrictions, this question was only asked among adolescents aged 14 or more (high school students).

#### Socio-Demographic Variables

Gender was built as a dummy variable with “girls” as the reference category. The Family Affluence Scale (FAS) (Currie et al., 2008) is an indicator of young people’s socio-economic status, comprised of six items on material assets in the family: ‘Does your family own a car, van or truck?’ [‘No’ (0), ‘Yes, one’ (1), and ‘Yes, two or more’ (2)], ‘Do you have your own bedroom?’ [‘No’ (0) and ‘Yes’ (1)], ‘During the past 12 months, how many times did you travel away on holiday with your family?’ [‘Not at all’ (0), ‘Once’ (1), ‘Twice’ (2), and ‘More than twice’ (3)], and ‘How many computers does your family own?’ [‘None’ (0), ‘One’ (1), ‘Two’ (2), and ‘More than two’ (3)] How many baths/showers are there in your house [None (1), one (2), two (3), and ‘more than two’ (4)], “Does your family have a dishwasher at home [no (1), yes (2)]. Reliabilities were α = 0.64 for the FSU – heritage, α = 0.56 for the Ethiopian-heritage group and α = 0.63 for the non-immigrant group. Scale scores were calculated by summing up the scores of all six items.

### Analysis

To examine our hypotheses, we used OLS linear regression to predict alcohol and cannabis use by each of the social capital measures (parental monitoring, friends support and teachers support) separately. Analysis included three non-interaction models [model 1- gender, age, family affluence, immigration group model 2- including perceived discrimination; model 3 – including measures of social capital (parental monitoring, friend and teacher support) and a fourth model including interactions between perceived discrimination and each of the social capital factors]. In order to examine the independent contribution of each of the measures of social capital, we then used OLS regression to test two models, in which alcohol and cannabis use were predicted in model 1 by the socio-demographic variables and all three social capital measures (parental monitoring, friend’s support and teachers support) and a second model including interactions between perceived discrimination and the three social capital measures. Due to the non-normal distribution of alcohol and cannabis use, we performed centralization of these variables using the Python application for SPSS software. Such transformation allowed us to use OLS analysis, which requires normal distribution of dependent variables.

## Results

Descriptive statistics of research variables are presented in **Table 1**. Alcohol use was significantly higher among FSU immigrants in comparison to the two other groups. Cannabis use among adolescents from FSU descent was significantly higher than cannabis use among non-immigrants. Levels of perceived discrimination were significantly different between all groups with Ethiopian adolescents reporting the highest and non-immigrants the lowest.

**Table 1:** Descriptive statistics of substance use, discrimination and social capital variables for Ethiopian, FSU and non-immigrant adolescents.

	Ethiopian immigrants	FSU immigrants	Non-immigrants	*F*	Range
	Mean (Standard deviation)	Mean (Standard deviation)	Mean (Standard deviatin)		
Alcohol use^a^	0.33 (0.88)	0.50 (1.00)	0.31 (0.77)	24.32^∗∗^	0–5
Cannabis use^b^	1.18 (0.88)	1.27 (1.00)	1.16 (0.79)	3.18^∗^	1–7
Discrimination^c^	1.90 (1.04)	1.56 (0.76)	1.31 (0.70)	163.17^∗∗^	1–5
Parental monitoring^c^	3.39 (0.56)	3.55 (0.47)	3.70 (0.39)	180.41^∗∗^	1–4
Friends’ support^c^	5.09 (1.60)	5.37 (1.36)	5.52 (1.36)	26.38^∗∗^	1–7
Teachers’ support^a^	3.85 (0.95)	3.72 (0.92)	3.88 (0.96)	10.24^∗∗^	1–5

*^∗^p < 0.05, ^∗∗^p < 0.01.*

*^*a*^Significant difference between FSU immigrant and two other groups.*

*^*b*^Significant difference between FSU immigrants and Jews non-immigrants.*

*^*c*^Significant difference between all groups.*

Additionally, **Table 1** shows the differences between ethnic groups in social capital recovery factors. Levels of parental monitoring were significantly different across all three groups with highest levels among non-immigrants and lowest levels among Ethiopian adolescents. The same significant pattern was found in perceived support of friends. Teachers’ support variable was significantly lower among FSU immigrants in comparison with the other two groups.

The correlations between research variables are presented in **Tables 2–4** for each national group separately. Among students from Ethiopian descent, in line with H1, alcohol use was positive associated with perceived discrimination and negatively related to all three social capital measures. Cannabis use was negatively related to parental monitoring. Among FSU adolescents (see **Table 3**), alcohol use was significantly positively related to perceived discrimination and negatively related to parental monitoring and teacher support. Cannabis use was also positively related to perceived discrimination and negatively related to parental monitoring. For non-immigrants (see **Table 3**), both alcohol and cannabis use were significantly positively related to perceived discrimination and negatively related to parental monitoring, friend and teacher support.

**Table 2:** Correlations between substance use, sociodemographic and social capital variables for Ethiopian adolescents.

	1	2	3	4	5	6	7	8
(1) Alcohol use (centered)	1							
(2) Cannabis use (centered)	0.45^∗∗a^	1						
(3) Gender (boys = 1)	0.18^∗∗^	0.18^∗∗a^	1					
(4) Age	0.23^∗∗^	–0.06^a^	0.03	1				
(5) FAS	0.05	0.33^∗∗a^	0.12^∗∗^	–0.09^∗^	1			
(6) Discrimination	0.18^∗∗^	0.14^a^	0.05	0.08	–0.06	1		
(7) Parental monitoring	–0.28^∗∗^	–0.20^∗a^	–0.13^∗∗^	–0.18^∗∗^	0.09^∗^	–0.14^∗∗^	1	
(8) Friends’ support	–0.25^∗∗^	–0.15^a^	–0.16^∗∗^	–0.09^∗^	0.05	–0.25^∗∗^	0.24^∗∗^	1
(9) Teachers’ support	–0.22^∗∗^	–0.02^a^	0.09^∗^	–0.05	0.00	–0.18^∗∗^	0.18^∗∗^	0.20^∗∗^

*^∗^p < 0.05, ^∗∗^p < 0.01.*

*^*a*^Students aged 15+ only.*

**Table 3:** Correlations between substance use, sociodemographic and social capital variables for FSU adolescents.

	1	2	3	4	5	6	7	8
(1) Alcohol use (centered)	1							
(2) Cannabis use (centered)	0.40^∗∗a^	1						
(3) Gender (boys = 1)	0.12^∗∗^	0.13^∗∗a^	1					
(4) Age	0.27^∗∗^	0.03^a^	–0.05	1				
(5) FAS	0.00	–0.10^∗a^	0.04	–0.03	1			
(6) Discrimination	0.16^∗∗^	0.16^∗∗a^	0.09^∗^	–0.05	–0.12^∗∗^	1		
(7) Parental monitoring	–0.28^∗∗^	–0.14^∗∗a^	–0.17^∗∗^	–0.10^∗∗^	0.09^∗∗^	–0.17^∗∗^	1	
(8) Friends’ support	–0.03	–0.01^a^	–0.17^∗∗^	0.06	0.11^∗∗^	–0.20^∗∗^	0.29^∗∗^	1
(9) Teachers’ support	–0.21^∗∗^	–0.06^a^	0.01	–0.10^∗∗^	–0.01	–0.26^∗∗^	0.27^∗∗^	0.20^∗∗^

*^∗^p < 0.05, ^∗∗^p < 0.01.*

*^*a*^Students aged 15+ only.*

**Table 4:** Correlations between substance use, sociodemographic and social capital variables for non-immigrant adolescents.

	1	2	3	4	5	6	7	8
(1) Alcohol use (centered)	1							
(2) Cannabis use (centered)	0.35^∗∗a^	1						
(3) Gender (boys = 1)	0.17^∗∗^	0.14^∗∗a^	1					
(4) Age	0.23^∗∗^	0.04^∗a^	–0.05^∗∗^	1				
(5) FAS	0.01	–0.04^a^	0.04^∗∗^	–0.05^∗∗^	1			
(6) Discrimination	0.09^∗∗^	0.16^∗∗a^	0.12^∗∗^	–0.07^∗∗^	–0.01	1		
(7) Parental monitoring	–0.22^∗∗^	–0.17^∗∗a^	–0.14^∗∗^	–0.08^∗∗^	0.07^∗∗^	–0.13^∗∗^	1	
(8) Friends’ support	–0.06^∗∗^	–0.09^∗∗a^	–0.20^∗∗^	0.10^∗∗^	0.09^∗∗^	–0.16^∗∗^	0.20^∗∗^	1
(9) Teachers’ support	–0.15^∗∗^	–0.09^∗∗a^	–0.02	–0.05^∗∗^	–0.02	–0.17^∗∗^	0.21^∗∗^	0.22^∗∗^

*^∗^p < 0.05, ^∗∗^p < 0.01.*

*^*a*^Students aged 15+ only.*

Results of the OLS regression analysis is presented in **Tables 5–8**. **Table 5** shows the association between parental monitoring and alcohol and cannabis use. Results showed that, in line with H1, perceived discrimination was positively associated with alcohol use and cannabis use. Additionally, parental monitoring, in line with H2, was negatively related to alcohol use and cannabis use. Also, in line with a recovery capital framework, and confirming H3, we found that parental monitoring had a moderating effect on the relationships between perceived discrimination and both alcohol and cannabis use. The association between friends’ support and substance use among adolescents is presented in the **Table 6**. In line with H2, there was negative association between alcohol and cannabis use and friend support. Hypothesis H3 for friends’ support was confirmed only partially: we found a moderating effect of friends’ support on the connection between perceived discrimination and risk behaviors only in the case of cannabis use. As expected, teachers’ support was negatively related to alcohol and cannabis use, in line with H2. Also, we found that teachers’ support (see **Table 7**), as hypothesized in H3, weakened the association between perceived discrimination and alcohol use. However, the interaction between perceived discrimination and teacher support was not significant for cannabis use.

**Table 5:** Regression analysis of the relationship between parental monitoring, discrimination and substance use.

	Alcohol use (centered)				Cannabis use (centered)			
	Model 1	Model 2	Model 3	Model 4	Model 1	Model 2	Model 3	Model 4
		
	β	β	*B*	β	*B*	β	β	β
Gender (boys = 1)	0.19^∗∗^	0.17^∗∗^	0.15^∗∗^	0.15^∗∗^	0.14^∗∗^	0.13^∗∗^	0.10^∗∗^	0.11^∗∗^
Age	0.24^∗∗^	0.25^∗∗^	0.23^∗∗^	0.23^∗∗^	0.04^∗^	0.05^∗∗^	0.05^∗∗^	0.05^∗∗^
FAS	0.03^∗^	0.03^∗∗^	0.05^∗^	0.05^∗∗^	–0.04	–0.03	–0.01	–0.02
Ethiopian immigrants	0.03^∗∗^	0.01	–0.02	–0.02	0.00	–0.03	–0.04^∗^	–0.05^∗^
FSU immigrants	0.06^∗∗^	0.05^∗∗^	0.04^∗∗^	0.04^∗∗^	0.04^∗^	0.02	0.01	0.01
Discrimination		0.11^∗∗^	0.08^∗∗^	0.43^∗∗^		0.15^∗∗^	0.13^∗∗^	0.38^∗∗^
Parental monitoring			–0.20^∗∗^	–0.11^∗∗^			–0.12^∗∗^	–0.05
Discrimination^∗^Parental monitoring				–0.35^∗∗^				–0.25^∗∗^
*R*^2^	0.10	0.11	0.14	0.15	0.03	0.05	0.06	0.06
*N*	6436				2752			

*^∗^p < 0.05, ^∗∗^p < 0.01. FAS, family affluence scale*.

**Table 6:** Regression analysis of the relationship between friend support, discrimination and substance use.

	Alcohol use (centered)				Cannabis use (centered)			
	Model 1	Model 2	Model 3	Model 4	Model 1	Model 2	Model 3	Model 4
		
	β	β	β	β	β	β	β	β
Gender (boys = 1)	0.19^∗∗^	0.17^∗∗^	0.17^∗∗^	0.17^∗∗^	0.14^∗∗^	0.13^∗∗^	0.11^∗∗^	0.12^∗∗^
Age	0.24^∗∗^	0.25^∗∗^	0.25^∗∗^	0.25^∗∗^	0.04^∗^	0.05^∗∗^	0.05^∗∗^	0.05^∗∗^
FAS	0.03^∗^	0.03^∗∗^	0.04^∗∗^	0.04^∗∗^	–0.04	–0.03	–0.03	–0.03
Ethiopian immigrants	0.03^∗∗^	0.01	0.01	0.01	0.00	–0.03	–0.04^∗^	–0.04^∗^
FSU immigrants	0.06^∗∗^	0.05^∗∗^	0.05^∗∗^	0.05^∗∗^	0.04^∗^	0.02	0.01	0.01
Discrimination		0.11^∗∗^	0.10^∗∗^	0.09^∗^		0.15^∗∗^	0.13^∗∗^	0.24^∗∗^
Friends’ support			–0.05^∗∗^	–0.06^∗∗^			–0.04^∗^	0.02
Discrimination^∗^Friends’ support				0.02				–1.96^∗^
*R*^2^	0.10	0.11	0.11	0.11	0.03	0.05	0.04	0.04
*N*	5946				2656			

*^∗^p < 0.05, ^∗∗^p < 0.01.*

**Table 7:** Regression analysis of the relationship between teacher support, discrimination and substance use.

	Alcohol use (centered)				Cannabis use (centered)			
	Model 1	Model 2	Model 3	Model 4	Model 1	Model 2	Model 3	Model 4
		
	β	β	β	β	β	β	β	β
Gender (boys = 1)	0.19^∗∗^	0.17^∗∗^	0.18^∗∗^	0.18^∗∗^	0.14^∗∗^	0.13^∗∗^	0.12^∗∗^	0.12^∗∗^
Age	0.24^∗∗^	0.25^∗∗^	0.23^∗∗^	0.23^∗∗^	0.04^∗^	0.05^∗∗^	0.05^∗∗^	0.05^∗∗^
FAS	0.03^∗^	0.03^∗∗^	0.03^∗^	0.03^∗^	–0.04	–0.03	–0.04^∗^	–0.04^∗^
Ethiopian immigrants	0.03^∗∗^	0.01	0.02	0.02	0.00	–0.03	–0.03	–0.03
FSU immigrants	0.06^∗∗^	0.05^∗∗^	0.04^∗∗^	0.04^∗∗^	0.04^∗^	0.02	0.01	0.01
Discrimination		0.11^∗∗^	0.09^∗∗^	0.22^∗∗^		0.15^∗∗^	0.12^∗∗^	0.15^∗∗^
Teachers’ support			–0.15^∗∗^	–0.08^∗∗^			–0.06^∗∗^	–0.04
Discrimination^∗^Teachers’ support				–0.14^∗∗^				–0.04
*R*^2^	0.10	0.11	0.13	0.13	0.03	0.05	0.04	0.04
*N*	5686				2552			

*^∗^p < 0.05, ^∗∗^p < 0.01.*

**Table 8:** Regression analysis of the relationship between social capital, discrimination and substance use.

	Alcohol use (centered)		Cannabis use (centered)	
	Model 1	Model 2	Model 1	Model 2
		
	β	β	β	β
Gender (boys = 1)	0.16**	0.16**	0.10**	0.10**
Age	0.22**	0.22**	0.05**	0.04*
FAS	0.04**	0.04**	–0.03	–0.03
Ethiopian immigrants	–0.01	–0.01	–0.05*	–0.05**
FSU immigrants	0.03*	0.03*	0.00	0.01
Discrimination	0.07**	0.42**	0.10**	0.44**
Parental monitoring	–0.18**	–0.10**	–0.12**	–0.05
Friends’ support	0.01	–0.02	–0.01	0.04
Teachers’ support	–0.11**	–0.07**	–0.04	–0.03
Discrimination^∗^Parental monitoring		–0.32**		–0.23*
Discrimination^∗^Friends’ support		0.07		–0.10
Discrimination^∗^Teachers’ support		–0.09*		–0.01
*R*^2^	0.16	0.16	0.05	0.06
*N*	5659		2538	

*^∗^p < 0.05, ^∗∗^p < 0.01. FAS, Family affluence scale.*

**FIGURE 2:**
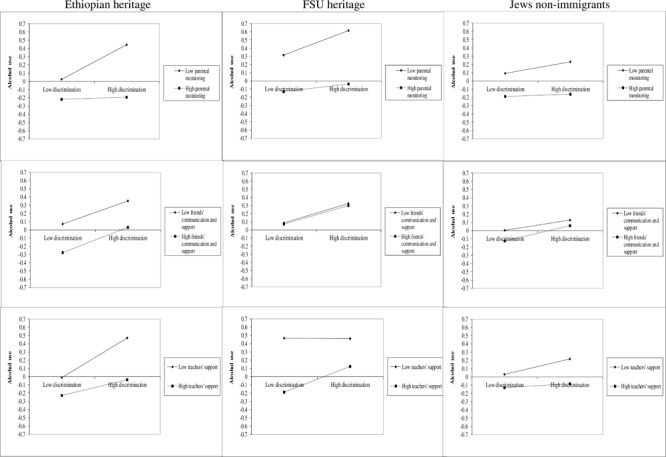
Interactions between discrimination, social capital and alcohol use by national origin.

**FIGURE 3:**
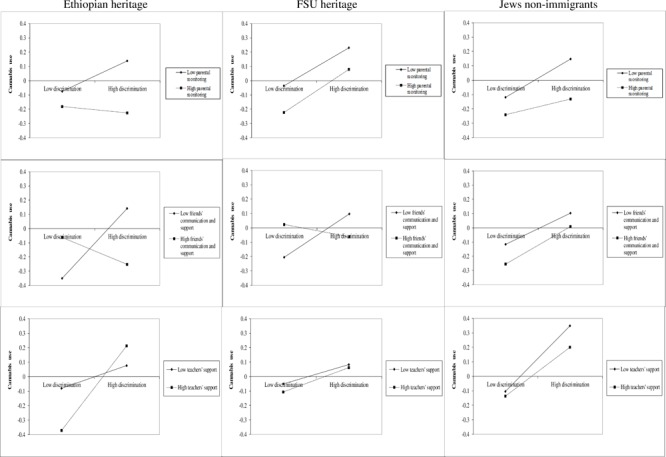
Interactions of discrimination, social capital and cannabis use by national origin.

**Table 8** shows the effect of all three measures of social capital the on association between perceived discrimination and alcohol and cannabis use. It can be seen that parental monitoring and teachers’ support were significantly negatively associated with alcohol use. The same pattern was found for interaction terms between these factors and perceived discrimination for parental monitoring and for teachers’ support. For cannabis use, only parental monitoring was found to be significant both as an independent predictor and as weakening the association between perceived discrimination and consumption of cannabis. Examination of the interactions across national origin (see **Figures 2, 3**) shows the strong positive relationship between perceived discrimination and alcohol and cannabis use, particularly in cases of low parental monitoring, across the three groups.

In addition, we examined the interaction terms between national descent, perceived discrimination and recovery social capital in order to understand whether there are differences in models for each group. We found only four significant interactions, three of which were with Ethiopian adolescents: between Ethiopian background, perceived discrimination and parental monitoring (β = 0.13, *p* < 0.05) and between FSU background, perceived discrimination and friends’ support (β = 0.13, *p* < 0.05) in prediction of alcohol use, and, also, between Ethiopian background, perceived discrimination and friends’ (β = -0.15, *p* < 0.05) and teachers’ (β = 0.18, *p* < 0.05) support in prediction of cannabis use. Examining the interaction graphs (**Figures 2, 3**), shows that for the Ethiopian adolescents there was a particularly strong relationship between perceived discrimination and alcohol use in the case of low parental monitoring, and between perceived discrimination and cannabis use in the case of low peer support and, surprisingly, high teacher support, as compared with non-immigrant adolescents.

## Discussion

The current study examined whether three elements of social capital (parental monitoring, friend and teacher support) moderate the association between perceived discrimination and substance use (problematic alcohol use and cannabis use) (Lorenzo-Blanco et al., 2017; Walsh et al., 2018) among immigrant and non-immigrant adolescents in Israel. Firstly, findings of the current study in which all three social capital measures were directly related to lower levels of substance use are in line with previous studies showing an association between parental monitoring (Gossrau-Breen et al., 2010; Thompson et al., 2015; Mynttinen et al., 2017), friend support (Wills et al., 2004) and teacher support (Walsh et al., 2010) and alcohol and cannabis use.

Yet, results go a stage further to suggest that social capital can moderate the relationship between perceived discrimination and substance use. Perceived discrimination can be considered a traumatic stressor (Carlson, 1997; Carter, 2007; Carter and Forsyth, 2010) in which feelings of interpersonal rejection and ostracism (Smart Richman and Leary, 2009) can lead to depletion of social and personal resources (Hobfoll, 1991) and high levels of psychological distress (Pascoe and Smart Richman, 2009) which may be expressed through substance use (Cooper et al., 1995). An RC paradigm suggests that levels of social capital can enable the individual to desist from substance use, due to the levels of support, empathy, caring and the social network which they provide (Connolly and Granfield, 2017). While little is written about RC within the developmental period of adolescence, in the current study, we extend RC to examine the relevance of social capital following trauma and stress and suggest that social capital, in the form of parental monitoring, friend and teacher support can enable the young person to process and make sense of the feelings of perceived discrimination, can enhance feelings of support, worth, belonging and care and counteract the messages from society that the young person is not wanted and has no future (Jasinskaja- Lahti et al., 2006). As such they may balance out the negative feelings that the young person may feel as a response to the perceived discrimination (Motti-Stefanidi et al., 2012). From a perspective of PTG (Calhoun and Tedeschi, 1999; Tedeschi and Calhoun, 2004), experiences of perceived discrimination can lead to negative schema related to the self (e.g., I have no value) and society (e.g., society doesn’t want me). Social capital can enable the building of adaptive narratives and schema, the experience of mutual support and empathy and feelings of belonging.

The findings around friend support are worthy of note. While past literature has tended to focus on levels of peer drinking and peer norms (Kuntsche and Jordan, 2006) as impacting on adolescent drinking, in this study we focused on friend support. In contrast with studies showing a positive relationship between peer drinking and alcohol use (Handren et al., 2016; Cambron et al., 2018) results here showed that friend support was negatively related to alcohol and cannabis use, although in the case of cannabis use there was a an interaction between friend support and perceived discrimination. In cases of low peer support there was a particularly strong relationship between perceived discrimination and cannabis use. Firstly, results stress that friends can play different roles as related to substance use. In line with a motivational model of alcohol use (Cooper et al., 1995), a self-medication explanation (Virtanen et al., 2015) or a stress-coping model (Wills and Cleary, 1995) results do show that friend support can be important in decreasing substance use. The difference between alcohol and cannabis use in relation to friends was notable. We suggest that the difference here between alcohol and cannabis use is that alcohol use is a more accepted, normative substance among peers (Sznitman et al., 2013) and, as such, peer support may not be so significant in reducing levels of use following perceived discrimination.

In the model in which all three social capital measures were entered found that, once the adult (parent and teacher) measures were entered, there were no significant relationships with friend support for either alcohol or cannabis use. In these cases it was parental monitoring (for both alcohol and cannabis use) and teacher support (for alcohol use) which were significantly related directly and as moderators of the perceived discrimination-substance use relationship. Findings also suggest that it may the adults, particularly parents, who can serve as the most powerful social capital in moderating the deleterious effects of perceived discrimination on young people. This is in line with previous studies showing the particular importance of the parental relationship in adolescent substance use (Wills et al., 1996, 2004). We hypothesis that since perceived discrimination may be perceived as a societal or institutional message of the extent to which a particular group is accepted (Jasinskaja- Lahti et al., 2006), the young person may be in need of an adult authority figure, to counterbalance the distress caused.

Immigrant groups showed lower levels of parental monitoring and peer support than non-immigrants and FSU adolescents showed lower levels of teacher support. Immigration places multiple stressors on a family (Conger et al., 2010; Cano et al., 2015a). Families need to cope with financial and occupational challenges (Leventhal and Brooks-Gunn, 2000), a loss of family and social networks, and the need to learn a new language, culture and norms (Torres et al., 2012). Intergenerational cultural dissonance (Kane et al., 2016) can make it hard for parents to monitor, particularly as their children may be resistant to accepting their parents values and norms. Immigrant adolescents may experience lower social support, due to the difficulties they have in fitting in and the sense of alienation and feeling different (Walsh et al., 2017). Immigrant adolescents often search for a peer group where they can feel a sense of strength and belonging (McNeely and Falci, 2004; Newman et al., 2007). Immigrant adolescents have also been found to find it hard to develop positive relations with agents of institutions (Zdun, 2011) such as teachers.

In addition, FSU adolescents, reported higher levels of alcohol use than the non-immigrant adolescents. Yet, in contrast with the hypothesis, Ethiopian heritage adolescents did not report greater alcohol use. Levels of FSU adolescents drinking may reflect socially accepted higher levels of alcohol drinking in Russian culture, as compared with Israeli culture (Hasin et al., 1998). The fact that levels of alcohol use among Ethiopian adolescents were not significantly higher than among Israeli born adolescents may challenge former studies pointing to higher levels of drinking among adolescent immigrants (Walsh et al., 2014). However, findings are in line with recent research showing that levels of drunkenness and heavy episodic drinking among adolescents may be related more strongly to levels of drinking in the country of origin (Barsties et al., 2017). Levels of alcohol use among the Ethiopian Jewish community were very low (Kebede et al., 2005; Grisaro and Witztum, 2012). However, Ethiopian adolescents did report higher levels of cannabis use which should be an issue of concern. Cannabis use has developmental implications (Tapert et al., 2008), on both current (Lynskey and Hall, 2000) and future (Fergusson and Boden, 2008) functioning and their higher levels of cannabis use may be a sign of inner distress.

As may be expected Ethiopian adolescents reported higher levels of perceived discrimination than FSU adolescents, who reported higher levels than non-immigrants. The difference in skin color and larger cultural gap can make Ethiopian adolescents more vulnerable to perceived discrimination both from the majority population and also from other immigrant groups (Ben-David and Ben-Ari, 1997; Ben-Eliezer, 2004). Yet, it is notable that the FSU adolescents also reported higher levels of perceived discrimination (Tartakovsky and Walsh, 2016) than non-immigrants.

### Limitations and Future Research Directions

The current study involves a representative sample of immigrant and non-immigrant adolescents in Israel. Yet, there are several notable limitations to the study. One major limitation is the cross-sectional, self-report nature of HBSC data. Longitudinal research designs would be needed to untangle the causal relationship between perceived discrimination, social capital and substance use. In addition, while validity has been questioned around adult retrospective reports of painful incidents in childhood (Hardt and Rutter, 2004) little is known about the validity of childhood self-reporting of perceived discrimination (Krieger et al., 2005). Retrospective self-reports may lead to the under-reporting of painful experiences such as perceived discrimination as well as possible exaggeration of them in order to justify the substance use. Some of the reliability coefficients were low, particularly in the case of FAS. The difficulties in assessing socio-economic status among children and adolescents are well-known and creating a measure which is valid across diverse cultural contexts is particularly difficult (Hartley et al., 2016). We were reluctant to leave out a socio-economic measure given its importance in the area of substance use, but acknowledge its problematic nature. The reliabilities for the Parental Monitoring Scale were also a little low for the Ethiopian and non-immigrant groups. Although the scale is a well-validated scale (Brown et al., 1993), it may be that historical changes in the nature of shared knowledge between parents and adolescents and/or cultural variations may limit the reliability of the scale. In addition, every immigration context is unique (Kwak, 2003). Israel is a particular immigration context and has been considered to encourage cultural assimilation amongst its immigrants (Ben-Eliezer, 2004). Further research is needed to explore in additional cultural contexts with additional immigrant groups, the relationship between perceived discrimination, social capital and substance use. In addition, we chose to use particular social capital variables due to their proven associations with substance use and due to their presence in the HBSC data. The HBSC study, while covering a large number of adolescent health behaviors, does not include variables which may be important for an understanding of adolescent substance use such as substance use specific communication (Reimuller et al., 2011) or levels of peer drinking (Simons-Morton et al., 2001). Due to the relatively small sample of Ethiopian adolescents, further research with a larger sample of respondents from Ethiopian descent is needed for a better understanding of the differences in mechanisms of influence of recovery social capital in this specific group. Lastly, due to the large sample size, some of the results are significant despite low power levels.

### Implications and Conclusion

Among its aims, RC provided a framework to assist clinicians and those working with substance users to desist from substance use (White and Cloud, 2008). The current study suggests that a framework of recovery capital can be useful for conceptualizing ways of moderating the negative impact of perceived discrimination. By strengthening parents abilities of monitoring their children in a new cultural environment and communicating with teachers as to their critical role in providing support, and balancing levels of perceived discrimination, levels of social capital may be able to counterbalance the stressful and potentially traumatic experience of perceived discrimination.

## Author Contributions

SW was responsible for conceptualizing the paper, overseeing the analysis, and writing the manuscript. TK was involved in conceptualizing the paper and was responsible for running the analysis. YH-F was the PI of the Israeli HBSC team and was involved in the conceptualization of the paper and editing of the manuscript.

## Conflict of Interest Statement

The authors declare that the research was conducted in the absence of any commercial or financial relationships that could be construed as a potential conflict of interest.

## Footnotes

1 Figures from the Israeli Central Bureau of Statistics, http://www.cbs.gov.il/shnaton63/st02_22x.pdf

2 The study focused only on these two immigrant groups as they are largest groups of immigrant adolescents in recent years to Israel. The sample included other small groups of heterogeneous immigrant groups (e.g., from the United States, South America, Morocco, and Romania). Since each group was too small for analysis and it did not make conceptual sense to combine them due to the large cultural differences, we focused on the two major groups.
